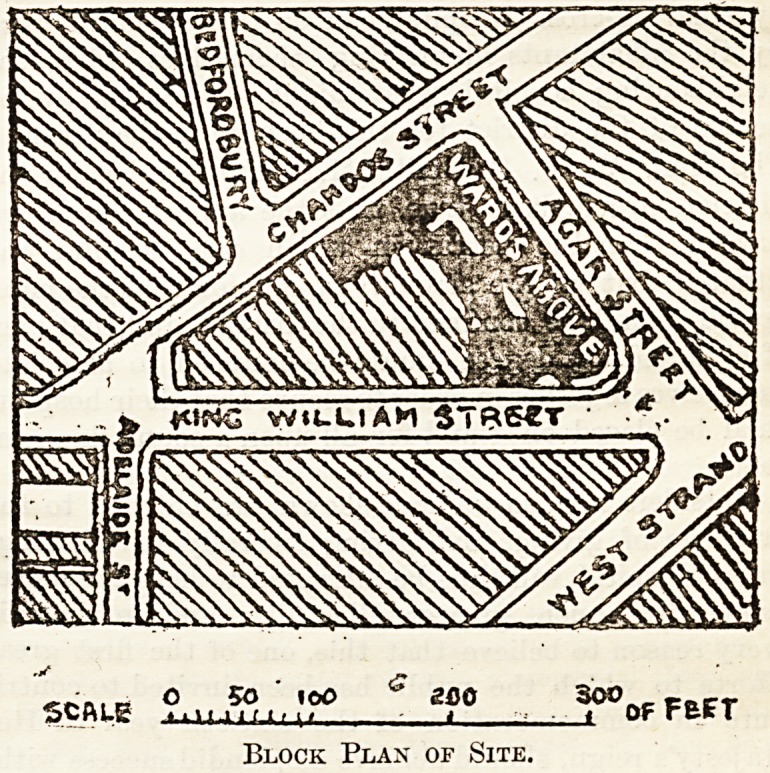# Within the Hospitals

**Published:** 1896-12-05

**Authors:** 


### The Institutional Workshop.

### WITHIN THE HOSPITALS.

### CHARING CROSS HOSPITAL,

It is an anomaly that Charing Cross Hospital, situated in the centre of what is probably the wealthiest district in the metropolis, should find itself, on the eve of the completion of the sixtieth year of Her Majesty's reign, in the condition described by one of the surgeons, Mr. J. Astley Bloxam, at the meeting on Saturday last. Owing to its central position the claims upon its resources have continuously increased year by year, but unfortunately the funds to meet those claims have proved altogether inadequate. Still, the work has to be done somehow, and Mr. Bloxam states: "The other day a scarlet fever case had to be put in the secretary's room for isolation, while at the same time an accident case and a furious delirium case were waiting beds. The space at present available was insufficient for the staff to carry on the essential duties of their profession. The nurses had no accommodation, and some of the resident medical officers who had no fixed emoluments had to sleep in the out-patients' department under such conditions that their lives were in danger." This state of affairs has been brought about in consequence of the Charing Cross Hospital having no endowment, and the impossibility of remedying evils of this kind without money, and plenty of money, too. Hence it is that a special committee has been appointed, not one moment too soon, whose duty it will be to commemorate the sixtieth year of the Queen's reign by raising £100,000 for the purposes of this hospital. No one who has the means to give, combined with the intelligence to grasp the facts of the situation, can properly hesitate to contribute liberally to this fund.

There will be many proposals and more suggestions to commemorate the year 1897. Several of the wealthiest men of the day are stated to be considering how they can fittirgly and wisely each expend a large sum in grateful recognition of the blessings received during the reign of Queen Victoria by the people of the British Empire. The first difficulty which confronts every man who has it in his power to give a really munificent sum is to select some site or some object which will secure that his commemoration shall stand prominently out as an object lesson and an encouragement to others for all time. The most suitable sites in the metropolis are already occupied, and land is so costly that the difficulty is thereby materially enhanced. We give a plan of the site of Charing Cross Hospital. It will be seen that it is self-contained; that is to say, that it is entirely surrounded by streets. As Lord Glenesk pointed out, the site is unique, for St. Paul's and Westminster Abbey look upon it from the east and the west, and there are the memories of the great and good there to stimulate them, while, if more is wanted, there are the ideas of Milton and duty and Gordon and Christian brotherhood emblematised close by, and speaking to them in eloquent words. Any building occupying the whole of this site adjacent to Charing Cross would face the Strand on the one side, St. Martin's Lane on another, and be within sight of Trafalgar Square. Beyond these considerations there is the further one that the committee have gradually acquired the whole, or nearly the whole, of this land, so that a millionaire, or the first millionaire who has the wisdom and the spirit to avail himself of the opportunity, for, to him, so relatively small a sum as £100,000, can secure at once, by associating himself with Charing Cross Hospital at this juncture, all the factors which are essential to make this individual commemoration of Her Majesty's reign an object lesson and a glorious memory for all time. The objects of the hospital are unsectarian; its benefits are universal; its teachings pregnant with good to the whole community. No more worthy or useful commemoration can be conceived than the one here offered to anyone who has the means and the brains to grasp it with gratitude. One hundred and seventy-five years ago Thomas Guy, the bookseller, gave more than twice £100,000 to establish Guy's Hospital, and has thereby perpetuated his memory and commended his judgment in the admiration and gratitude of Englishmen for ever. When we remember, as has been recently stated in the papers, that by one transaction a millionaire has recently made £500,000 in a few weeks, it should not be too much to hope that more than one of the several men who have it in their power will compete for the honour of following in Thomas Guy's footsteps by taking Charing Cross Hospital under his wing.

Block Plan of Site.

Apart from millionaires, the site affords a further text upon which to base an argument which should secure more than all the money required within the next few months. The district surrounding Charing Cross Hospital is probably as wealthy as any district of London. This hospital is the hospital of the district first of all. It provides for the needs of the sick and suffering poor within its boundaries, though in addition to this it has a claim as the great accident hospital of Central London upon all whose material wealth is in any way contributed to by the resources the metropolis affords. If the committee can once succeed in inducing the busy inhabitants, and those wealthier and more fortunate people who have their places of business in the district but reside elsewhere, to look the needs and claims of Charing Cross Hospital fairly in the face, we make no doubt that not only will the £100,000 required be forthcoming, but that its annual income will be raised to a sufficient sum to enable the work to be continued with the maximum of efficiency henceforward. London hospitals, or many of them, have suffered in the past from the fact that all have appealed mainly to the same class of people, whilst most of them have neglected to identify their institution with the district in which it is placed. One notable exception to this short-sighted system is afforded by the Great Northern Central Hospital, the promoters of which had the courage about fourteen years ago to select a site in the heart of North London, to build upon it a hospital of surpassing excellence, and to expend upon that work a sum which approached £100,000. Not only has all this money been forthcoming mainly from the contributions of the inhabitants of North London, but the permanent ordinary income has been raised from the same source from £3,700 to upwards of £8,000 per annum. The invested funds, too, show considerable development, and are steadily increasing. If North London, which has not a tithe probably of the wealth of the district served by Charing-cross Hospital, can do this splendid work by the inhabitants identifying themselves with the neighbouring hospital, what might not be done by the people of the district for this hospital in the Strand? The Hon. W. F. D. Smith, M.P., the member for the district, urged, it is true, that the appeal should go further than the district in which the hospital was situated, but we are strongly of opinion that the main appeal should be confined to the district, and that men of high and low degree should be induced to associate themselves together in order to secure that their hospital shall be placed once and for all upon a sound financial basis.

Outsiders who, as we have shown, are entitled to the privilege of giving, and giving liberally to Charing-Cross Hospital, will thus be attracted to help the movement with much greater liberality, and so there is every reason to believe that this, one of the first great efforts to which the public has been invited to contribute in commemoration of the sixtieth year of Her Majesty's reign, should achieve a splendid success without difficulty or delay. Hospitals are not institutions which can properly descend to importunate begging. Their splendid record, the services they render to every man and woman in the land, and the imperative necessity to all classes for their existence and for their efficient maintenance, speak to every right feeling Englishman and Englishwoman with an eloquence and a power which no mere words could equal. What Charing Cross Hospital has lacked in the past has been a voice and an energetic persistence in arousing and fixing the attention of the inhabitants of the district and of all Londoners to the splendid opportunity it presents to one and all of them to give of their substance, and to give liberally, knowing that the money they may thus contribute will avail to effect in the best way the maximum of good for a large number of unfortunate people who have been striken by Providence with accident or disease.

We venture to hope that our colleagues in the press may be able to find space to bring out these considerations, and to drive them home to the consciences of the people of all classes.

### CHARING CROSS HOSPITAL.

### Meeting of the Special Appeal Committee.

At a special meeting of the Council of Charing Cross Hospital held in July last, H.R.H. the Duke of Saxe-Coburg and Gotha, K.G., president, in the chair, it was determined to make a strenuous effort to raise the sum of £100,000 in order to place the hospital in a sound financial condition and to carry out improvements necessary to its efficiency. A meeting of the Special Appeal Committee appointed with this object was held in the board-room of the hospital on the 28th ult. under the presidency of the Duke of Saxe-Coburg and Gotha.

The Chairman, who was greeted with applause, expressed on behalf of himself and of those connected with the hospital the great pleasure he felt at the large number present. Charing Cross Hospital had done a great deal of good for many years, and the real necessities of the case were well shown in the last paragraph in the appeal, which ran as follows: "The situation of Charing Cross Hospital is unique. The enormous traffic of the Strand and of the adjacent thoroughfares causes a large number of street accidents which require to be dealt with on the spot. There is a large and poor population resident in the immediate neighbourhood. The demand upon the accommodation of the hospital is constantly increasing. The present income is hopelessly inadequate. The hospital has no reserve fund, and its buildings are heavily mortgaged. It follows that Charing Cross Hospital cannot be carried on unless a generous response is made to this appeal." In conclusion, he expressed a hope that all who were present would do their best to ensure the success of the appeal. (Applause.)

Letters regretting their inability to attend were read from Mr. J. B. Martin (treasurer), Mr. Henry C. Burdett, Sir Whittaker Ellis, and Sir Stuart Knill.

Lord Glenesk, in moving the following resolution, "That this meeting fully recognises the great services rendered to the public by Charing Cross Hospital, during the 75 years of its existence, and emphatically declares its opinion that the hospital should be generously supported, and maintained in its unique position," said that the most eloquent appeal which could be made on an occasion like the present one was made by absolute and true facts. Charing Cross Hospital, during the 75 years of its existence, had discharged a great public work. Unfortunately, however, the hospital had not increased with the increasing needs of the neighbourhood, and the result was that one of the main objects of the appeal was to ask for more room. As an instance of the increased work the speaker said that 20 years ago 1,181 in-patients, 14,900 out-patients, and 4,153 accident cases were treated in the course of a year; in 1895 the numbers were 2,100 in, 22,800 out-patients, and 11,280 accident cases.

Mr. C. H. Combe, M.P., seconded, and spoke of the absolute necessity of having the hospital on the site which it at present occupied. He had heard, with a considerable amount of astonishment, that a suggestion had been made to move the hospital, and this, he hoped, would remain a suggestion, for, in his opinion, the present site was an ideal one. It was situated in a central position, near great centres of traffic, and in the midst of a teeming population, all of which would be the chief points borne in mind were one selecting a site on which to build a new hospital.

The resolution was carried unanimously.

Mr. J. Astley Bloxam moved, "That the usefulness of the hospital is seriously crippled in its work by want of adequate accommodation, and that additional buildings are urgently necessary to meet the want." In support of this resolution, he emphasised the fact that though during the last 20 years the patients, as Lord Glenesk had shown, had greatly increased, the accommodation had remained stationary. The increase in patients, and the advances made by surgery and medicine, had rendered a new out-patients' department a necessity. The present out-patients' department was so crowded as to be almost unbearable. It was almost a case of Box and Cox—(laughter)—no sooner was one class of case out than another class was let in. New wards for the reception of special cases were also necessary, as the following instance would show. The other day, when every ward in the hospital was engaged, when even the hall was occupied by a case rendered insensible from a scaffolding accident, a poor woman brought in a child suffering from scarlet fever. There was absolutely nowhere to put the case except in the secretary's office, and there it was at last placed. That would show how crowded the hospital was—in fact there was absolutely not enough room for the staff to carry on the essential duties of their profession. There was no accommodation for offensive diseases, or for those diseases which required isolation. In addition the hospital wanted a nursing home, and accommodation for the resident medical staff. In regard to the latter requirement, Mr. Bloxam mentioned that some of the resident staff had to sleep in the out-patient department, which had been crowded all day with patients.

Dr. Mitchell Bruce seconded, and pointed out that there was no proposal to extend the general wards of the hospital; all that was wanted was merely to provide room for the patients who came to them for treatment. What they asked for were matters of necessity—the means of being efficient. It was a very sorry confession to make, but the work was not as well done as it might be, because there was neither the accommodation nor the means to treat the patients as they ought to be treated.

The resolution was carried.

The Hon. W. F. D. Smith, M.P., moved, and Sir Charles Hall, K.C.M.G., Q.C., M.P. (Recorder of London), seconded, the following resolution, which was carried unanimously: "That for the purpose of maintaining this hospital, and extending its usefulness, it is imperative that a special appeal should be made, and this meeting pledges itself to use every effort on behalf of the appeal for £100,000 to be raised in commemoration of Her Majesty's reign."

A vote of thanks to the Chairman, proposed by Mr. G. J. Drummond (treasurer of the hospital), seconded by Lord Wantage, K.C.B., V.C., closed the meeting.

## Figures and Tables

**Figure f1:**